# Acetylated Tau Protein: A New Piece in the Puzzle between Brain Ischemia and Alzheimer’s Disease

**DOI:** 10.3390/ijms23169174

**Published:** 2022-08-16

**Authors:** Ryszard Pluta, Sławomir Januszewski, Mirosław Jabłoński

**Affiliations:** 1 Laboratory of Ischemic and Neurodegenerative Brain Research, Mossakowski Medical Research Institute, Polish Academy of Sciences, 02-106 Warsaw, Poland; 2 Department of Rehabilitation and Orthopedics, Medical University of Lublin, 20-090 Lublin, Poland

Cerebral ischemia in humans and animals is a life-threatening neuropathological event and leads to the development of dementia with the Alzheimer’s disease phenotype [1]. Brain ischemia can also initiate or accelerate progressive neurodegenerative processes and is a known epidemiological risk factor for Alzheimer’s disease [2,3,4,5]. First, both ischemic stroke and Alzheimer’s disease share the same risk factors [6]. Second, post-ischemic death of neurons is directly mediated by excitotoxicity [7] and indirectly by the neurotoxicity of folding proteins [8,9]. Third, post-ischemic brain studies have shown the breakdown of the blood–brain barrier as in Alzheimer’s disease [10,11,12,13,14]. Fourth, post-ischemic oxidative stress violates the integrity of the genome, causing DNA damage; death of neuronal, glial, and vascular cells; and neurological deficits following cerebral ischemia [15]. Fifth, in neurodegeneration after ischemia, a lack of acetylcholine in the brain has been noted, as in Alzheimer’s disease, especially in the hippocampus, acetylcholine is an essential neurotransmitter that plays a key role in synaptic transmission and memory formation, and a lack of it is a significant cause of dementia [16]. Sixth, the reduction in the length of brain vessels after ischemia or their spasm impairs cerebral blood flow [17]. Seventh, after cerebral ischemia, dominant neuronal death in the hippocampus is observed, with the development of a general brain atrophy identical to Alzheimer’s disease [1,18,19,20]. Eighth, the neuroinflammatory response plays an important role in the progression of post-ischemic neurodegeneration, such as in Alzheimer’s disease [21,22,23]. Ninth, data show that in post-ischemic brain, Alzheimer’s disease-specific folding proteins such as amyloid and tau protein are induced, leading to the induction of amyloid plaques and neurofibrillary tangles [1]. Tenth, both diseases develop cerebral amyloid angiopathy [24]. Eleventh, dysfunction of the mitophagy, autophagy, and apoptotic genes is engaged in post-ischemic neurodegenerative processes, such as in Alzheimer’s disease [1,25]. Twelfth, an ischemic episode of the brain accelerates the onset of dementia by 10 years, and 10% of survivors develop dementia soon after the first and 41% after recurrent ischemic stroke [24,26]. Thirteenth, observations indicate that the cerebral ischemic episode is the causative initiator of Alzheimer’s disease [1,2,3,4,5,20]. Post-ischemic neurotoxic mechanisms generated by amyloid and the tau protein are believed to play a key role in the development of irreversible post-ischemic neurodegeneration [1,4,5,20]. Alzheimer’s disease-related proteins, such as amyloid and tau protein, and their genes are believed to play a fundamental role in post-ischemic neuron death and progressive neurodegeneration.

There are currently no treatments that can prevent progressive ischemic damage and delay or stop the post-ischemic neurodegenerative processes that are dependent on amyloid and tau protein. In the absence of translational experimental cerebral ischemia therapies for clinical use, the focus is now on reducing the neurotoxic effects of amyloid and tau protein on post-ischemic neuronal cells in order to prevent progressive neurodegeneration of the brain with the development of Alzheimer’s disease phenotype dementia. There is growing awareness and a realization of the challenges faced by different countries as the proportion of population living with symptoms of dementia continues to grow. Despite a large amount of research into the causes and processes leading to dementia and numerous projects to look for therapies modifying the progress of dementia that will have a decisive impact on neurodegeneration after ischemia and the development of Alzheimer’s disease, there has still been no breakthrough [27,28,29]. Moreover, drug failures affecting the progression of dementia are not optimistic at the moment. Therefore, it is essential to finally understand the underlying processes to think about developing new therapeutic approaches. Ischemic brain damage and Alzheimer’s disease lead to the death of neurons, which drastically reduces their number in the neuronal network, adversely affecting the elderly who already have a reduced number of neurons due to aging. Due to the lack of causal treatment of brain ischemia and Alzheimer’s disease [27,28,29], it is advisable to investigate, compare and search for similarities in the development and pathological processes occurring in both neuropathological units. This is particularly relevant when new data indicate that ischemic mechanisms may take part in the development of Alzheimer’s disease and that there is a similarity of molecular phenomena between brain post-ischemic neurodegeneration and Alzheimer’s disease [1,3,4,5].

Recent data show an association between the CA1 and CA3 hippocampal regions and changes in the expression of the tau protein gene within 2, 7 and 30 days after ischemia [30,31]. In the CA1 region, expression of the tau protein gene increased significantly on the second day post-ischemia (Table 1) [30]. However, the expression of this gene 7–30 days after ischemia (Table 1) fluctuated around the control level [30]. On the other hand, in the CA3 region, fluctuation about the control value of the tau protein gene expression was noted on the second day after ischemia (Table 1) [31]. However, the expression of the significant growth of the tau protein gene was noted 7–30 days post-ischemia (Table 1) [31].

Increased accumulation of tau protein in neuronal cells, astrocytes and oligodendrocytes in the cortex, hippocampus and thalamus has been established in experimental studies [32,33,34,35,36,37] as well as in human brains after ischemia [38,39,40]. Accumulating tau protein was also visible in the microglia in the focal brain ischemia penumbra [40,41,42,43]. Observations indicate that brain cells accumulate the tau protein as a result of ischemia [35], which indicates a significant phase in the development of pathological ischemic processes in the brain tissue [36]. The next study found that the modified tau protein blocks the traffic of neurofilaments, organelles, amyloid protein precursor vesicles and rises oxidative stress in the neuronal body, axons and dendrites, leading to the accumulation of amyloid protein precursor in neurons [44]. To this must be added the increase in the level of total tau protein that was investigated by brain microdialysis after complete ischemia [45].

It is currently believed that cerebral ischemia causes and/or accelerates the age of onset of Alzheimer’s disease [2]. Alzheimer’s disease neuropathology is observed in patients with post-ischemic stroke dementia. However, our understanding of the pathology of both disease entities remains limited due to the lack of individual characteristic elements in the pathological puzzle, e.g., in the development of neurofibrillary tangles after cerebral ischemia, which makes therapeutic targets elusive. An article in the *International Journal of Molecular Sciences* presented a previously unknown molecular factor associated with the modification of the tau protein, which at the same time strongly supports the relationship between cerebral ischemia and Alzheimer’s disease [46]. This new missing factor is the acetylation of the tau protein, which is a significant phenomenon in Alzheimer’s disease [46]. Adding acetylated tau protein to the puzzle between cerebral ischemia and Alzheimer’s disease brings us one step closer to possible prevention and/or treatment of dementia after ischemia and neurodegenerative disorders.

The following are the levels of phosphorylation and acetylation of tau protein and tau protein associated proteins in the cerebral cortex in the ischemic group on the ipsilateral cortex and contralateral cortex [46]. The levels of phosphorylated tau protein and acetylated tau protein in ipsilateral cortex significantly increased compared to the contralateral cortex [46]. Phosphorylated glycogen synthase kinase 3-beta was not significantly different between contralateral cortex and ipsilateral cortex [46]. The level of sirtuin 1 was also not different between ipsilateral cortex and contralateral cortex [46].

The following are the levels of phosphorylation and acetylation of tau protein and tau-protein-associated proteins in the cerebral cortex in the ischemic rehabilitation group on the ipsilateral cortex and contralateral cortex [46]. The expression levels of phosphorylated tau protein in ipsilateral cortex were not significantly different compared to contralateral cortex; however, the level of acetylated tau protein significantly increased in the ipsilateral cortex. The level of phosphorylated glycogen synthase kinase 3-beta was not significantly different between the ipsilateral cortex and contralateral cortex. The level of sirtuin 1 was not significantly different between the ipsilateral cortex and contralateral cortex either [46].

The following are the levels of phosphorylation and acetylation of tau protein and tau-protein-associated proteins in the cerebral cortex in the ischemic rehabilitation group and in the ischemic group on the ipsilateral cortex after three months of aerobic exercise [46]. The level of acetylated tau protein in the ipsilateral cortex was significantly lower in the rehabilitation group. The level of phosphorylated glycogen synthase kinase 3-beta was lower in the rehabilitation group. Sirtuin 1 significantly decreased in the ipsilateral cortex in rehabilitated animals. Additionally, aerobic training improved balance, motor and memory functions. Rehabilitation with aerobic exercise inhibited tau protein acetylation after focal brain ischemia, which was accompanied by the improvement of motor and cognitive functions [46].

In this study, no difference in tau protein phosphorylation was found between the ischemic and ischemic rehabilitation groups. Looking at the results, further studies in a larger group of animals are warranted, and the study of the tau protein in a specific region, such as the hippocampus, which is dominated by changes in cerebral ischemia and Alzheimer’s disease. In another pathological component of tau protein pathogenesis, acetylated tau protein was significantly lower in the ipsilateral cortex in the ischemic rehabilitation group compared to the ischemic group [46]. Acetylated tau protein promotes tau protein hyperphosphorylation, aggregation and the formation of neurofibrillary tangles (Figure 1). This is the first study to show a protective role of aerobic training in post-ischemia tau protein-associated neurodegeneration. Sirtuin 1 was found to reduce the acetylation of the tau protein [47]. However, the inhibitory effect of aerobic training on tau protein acetylation appears to be independent of the sirtuin 1 mediated mechanism, as ipsilateral cortex sirtuin 1 levels in the ischemic rehabilitation group were significantly lower than in the ischemic group [46]. The inhibitory effect of aerobic training on tau protein acetylation is likely due to other unknown acetylation and deacetylation mechanisms. When the ipsilateral cortex was compared to the contralateral cortex, elevated levels of acetylated tau protein were observed in the ipsilateral cortex in both the ischemic and ischemic rehabilitation groups. This suggests that the pathology of the tau protein due to acetylation, followed by a neurotoxic effect, can be sustained even after three months of aerobic training. Therefore, given the phosphorylation of tau protein, the prolonged action of acetylated tau protein through reperfusion should be a therapeutic target after stroke.

Reversible local ischemic brain injury with 1-day rat survival causes specific hyperphosphorylation of the tau protein near the site of the injury [41]. In the case of the death of pyramidal neurons in the CA1 area of the hippocampus post-ischemia in gerbils, hyperphosphorylation in serine 199/202 of the tau protein is controlled by mitogen-activated protein kinase, cyclin-dependent kinase 5 and glycogen synthase kinase 3 [48]. Studies have shown that post-ischemia, the tau protein undergoes hyperphosphorylation in neurons and is closely related to the development of apoptosis [41,42,43,49,50,51]. Hyperphosphorylation results in the formation of paired helical filaments after brain ischemia [52], neurofibrillary tangle-like [49,50,51] and neurofibrillary tangles [53,54] characteristic of Alzheimer’s disease (Figure 1). In support of the above phenomena, elevated levels of cyclin-dependent kinase 5, directly involved in the development of neurofibrillary tangles, were noted after experimental post-ischemic brain injury [51].

The increase in tau protein has been noted in the serum after complete cerebral ischemia, which is associated with progressive damage to neurons during recirculation [55]. These data indicate that serum tau protein levels may be a prognostic indicator of post-ischemic brain damage [55,56]. Increases in tau protein levels have also been demonstrated in the serum after ischemic stroke in humans, and it appears to be an indicator of the progression of damage to neurons and their axons after ischemia [57,58,59,60,61]. An increase in the level of tau protein in the cerebrospinal fluid of patients after ischemic stroke has also been documented [61]. Increase in blood and cerebrospinal fluid tau protein after ischemia negatively correlates with clinical outcomes.

By itself, ischemia of the brain causes an increased permeability of the blood–brain barrier [10,11,12,13,14], which influences hyperphosphorylation of the tau protein [41,42,43,49,51,52,53,54,62,63], and the pathological tau protein causes additional damage to the blood–brain barrier, which induces harmful feedback [64]. Post-ischemic accumulation of amyloid in the brain associated with increased permeability of the blood–brain barrier [65,66,67,68,69,70,71] enables the onset of tau protein dysfunction, which supports the link between amyloid accumulation and tau protein modification at some stage of damage to the blood–brain barrier [64]. Moreover, both oxidative stress [72] and neuroinflammation [21,22,23] cause damage to the blood–brain barrier that may cause hyperphosphorylation of the tau protein and the development of neurofibrillary tangles post-ischemia (Figure 1) [51,53,54,73]. Moreover, post-ischemia, the plasma-derived tau protein [55,56] crosses the post-ischemic blood–brain barrier in two directions and may exacerbate its own pathology in the brain tissue [74]. Post-ischemic insufficiency of the blood–brain barrier exacerbates the pathology of the tau protein in the brain and also indicates that ischemic pathology of the brain is the cause of the increase in the concentration of tau protein in the serum and in the cerebrospinal fluid [55,56,74,75].

Alzheimer’s disease is the most widespread form of dementia, accounting for 60–80% of all dementia cases [76]. The disease affects more than 40 million patients worldwide, and the incidence of Alzheimer’s disease doubles every five years after the age of 65 [77]. The established failure of anti-amyloid therapy with the intention of changing the course of Alzheimer’s disease in clinical trials has led to a radical change in thinking about the etiopathogenesis of the disease [27,28,29]. This situation has directed a group of scientists to brain ischemia, which, like Alzheimer’s disease, mainly has changes in the hippocampus. Post-ischemic brain damage is a worldwide health problem that affects more than 40 million people each year. Post-ischemic brain injury causes cognitive impairment in approximately 70% of patients, is the third most common cause of disability, the second most common cause of dementia in the world, and may soon become the leading cause of dementia according to the latest forecasts [18,78]. Looking at the period 1990–2016, the average global lifetime risk of ischemic stroke increased by approximately 9% [79].

An article in the *International Journal of Molecular Sciences* reported that two forms of post-translational modifications of the tau protein, nitrosylation and acetylation, can fuel post-ischemic brain damage [46]. Meanwhile, microtubule-associated tau protein has been shown to be a major component of neurofibrillary tangles in Alzheimer’s disease as well as in cerebral ischemia [47,53,54]. Tauopathy, which can also include brain ischemia, is closely related to abnormal posttranslational modifications of the tau protein, including acetylation, phosphorylation and ubiquitination, which alter the stability of microtubules, reduce the solubility of the tau protein and promote the formation of tau protein aggregates [80]. Although acetylation of the tau protein is known to be a major player in neurodegeneration, only recently have more acetylation sites been identified [81] than the phosphorylation and ubiquitination sites combined [82]. In the study of Mankhong et al. [46], for the first time, a long-lasting increase in acetylation of the tau protein in the brain cortex after ischemia was observed. They also found that hyper-acetylation on the tau protein leads to decrease activity of sirtuin 1 [46]. This finding is in line with clinical research suggesting that acetylation of the human tau protein is a promising new pathological biomarker in Alzheimer’s disease [82].

During neurodegenerative changes, an increase in reactive forms of nitrogen and nitrosative stress was observed [83]. Abnormal S-nitrosylation of proteins also occurs in post-ischemic neurons [15]. Current research indicates that S-nitrosylated GAPDH not only activates two closely related acetyltransferases, p300 and the CREB binding protein [84], but also mediates S-nitrosylation of sirtuin 1 and inhibits its deacetylase activity [47]. Thus, the balance between tau protein acetylation by p300/CREB binding protein and deacetylation by sirtuin 1 is completely tilted after ischemia, resulting in pathogenic and long-term acetylation of tau protein in neurons.

Brain ischemia is an acknowledged key age-related environmental risk factor for Alzheimer’s disease [2,3,4,5]. Post-ischemic events related to recirculation are highly interactive during prolonged survival and can result in serious and life-long complications in survivors of cerebral ischemia. Neuronal proteinopathies associated with amyloid, tau protein, and α-synuclein have been consistently reported in ischemic patients and in pre-clinical ischemic brain studies. The current data that acetylated tau protein has a significant impact on post-ischemic neurodegeneration [46] not only open a new direction of diagnosis but also provide an opportunity to thoroughly investigate the convergent mechanisms between brain ischemia and Alzheimer’s disease. Therefore, it is also suggested that acetylated tau protein in serum may be a novel and reliable indicator of post-ischemic damage that may be linked to amyloid and tau protein [55,56,57,58,59,60,61,69,70]. The novelty of the current study may not only be the discovery of signaling pathways that drive tau protein acetylation, but also supporting the possibility of innovative therapy through rehabilitation after ischemia [46].

In addition to the tau protein, there are other mechanisms underlying the association of cerebral ischemia with Alzheimer’s disease, including amyloidosis [47], neuroinflammation [21,22,23] and cerebral amyloid angiopathy [24]. Increased levels of serum amyloid and tau protein as well as acetylated tau protein indicate that the blood–brain barrier shows increased permeability, as in Alzheimer’s disease [64]. Therefore, the integrity of the blood–brain barrier should be taken into account when assessing the role of acetylated tau protein, post-ischemic outcomes, and the events underlying neurodegeneration and dementia, and in determining timing and treatment routes. By adding acetylated tau protein to this puzzle between ischemic brain injury and Alzheimer’s disease, we are one step closer to possibly stopping and/or preventing the development of Alzheimer’s disease and post-ischemic neurogenerative disease.

Alzheimer’s disease and cerebral ischemia are characterized by the accumulation of tau protein in brain tissue, which is associated with loss of synapses and cognitive decline. The discussed article from the *International Journal of Molecular Sciences* highlights the abnormal acetylation of tau protein as a key to understanding the pathophysiological role of the protein in post-ischemic neurodegeneration; this is precisely what has been lacking so far [46]. Specific acetylation sites regulate tau protein aggregate formation, synaptic signaling, and long-term potentiation [47]. Discovering more details about the history associated with tau protein acetylation could provide new insight into potential therapeutic approaches to debilitating neurodegenerative diseases such as cerebral ischemia and Alzheimer’s disease.

New research suggests that the acetylation of soluble tau protein may be an early stage of the disease, and inhibiting this process, e.g., by aerobic exercise, may be a potential therapeutic strategy [46,47]. However, recent evidence shows that tau protein fibrils are not the pathogenic culprit and points to soluble tau protein [85]. Acetylation of soluble tau protein has a significant impact on its properties, including its stability and aggregation, and the level of acetylated tau protein increases in the brain in Alzheimer’s disease and after ischemia [46,47]. In a study by Min et al. [86], a possible link between tau protein acetylation and Alzheimer’s disease was investigated. They showed that the residue of lysine 174 (K174) is acetylated in the brain with Alzheimer’s disease. They then showed that acetylation decreases the turnover of the tau protein. The question is: what is the final effect of acetylated tau protein on brain tissue? In mice, overexpression of the mutant K174Q tau protein in the hippocampus led to a much greater atrophy in this area at 3 months than overexpression of the wild-type tau protein. In addition, K174Q overexpressing mice performed worse on spatial learning and memory retention tests. Finally, they investigated the effect of inhibiting the activity of p300, which is the acetyltransferase responsible for the acetylation of the tau protein. In an in vitro study, the p300 inhibitor salicylate reduced the levels of acetylated tau protein in K174 and increased tau protein turnover. The data presented highlight the acetylation of tau protein as an important pathogenic stage in Alzheimer’s disease and cerebral ischemia and suggest a new therapeutic approach for these currently incurable diseases.

Depending on the acetylated lysine, acetylation of the tau protein has an impact on it properties, including tau protein turnover [87], synaptic plasticity [88], missorting [89], phosphorylation [90] and aggregation [91]. Protein deacetylase sirtuin 1 has been shown to reduce acetylation of tau protein in a mouse model of neurodegeneration [87]. It has been shown that sirtuin 1 deficiency in the brain increases synaptic disappearance and behavioral deficits, and sirtuin 1 overexpression alleviates the spread of tau protein pathology [87]. Excessive behavioral deficits and premature death were found in mice after sirtuin 1 was removed from the brain in a model of neurodegeneration [87]. In contrast, injection of AAV-sirtuin 1 into the hippocampus decreased the levels of acetylated K174 tau protein and limited the spread of the pathogenic tau protein in the same experimental model [87]. Brain studies with Alzheimer’s disease revealed the shared localization of acetylated tau protein at the 280 (K280) lysine residue with phosphorylated tau protein in neurofibrillary tangles [87]. Increased amounts of acetylated neurofibrillary tangles K280 were directly related to more advanced stages of Alzheimer’s disease [87]. Taken together, this evidence suggests that tau protein deacetylation may be a reliable strategy to inhibit tau protein aggregation and propagation in cerebral ischemia and Alzheimer’s disease. One important way to reduce acetylated tau protein is by targeting sirtuins. Sirtuin 1 may deacetylate acetylated tau protein, especially in Alzheimer’s disease and cerebral ischemia, the levels of which gradually decline as both diseases progress [46,87]. This is confirmed by data from Mankhong et al. [46]. It has been shown that sirtuin 1 can deacetylate tau protein residues 160–182 and 264–287 in vitro [87]. Additionally, sirtuin 1 deacetylates units of the autophagy process, which may lead to increased removal of intracellular aggregates of the tau protein [92].

Acetylation of the amino acid lysine in the tau protein has been found to prevent ubiquitin-mediated degradation, resulting in neurofibrillary tangles similar to those found in dementia [93]. Moreover, it has been suggested that the hyperacetylation of lysine in the tau protein contributes to the accumulation of β-amyloid peptide in Alzheimer’s disease and to cognitive impairment, indicating that such a mechanism may also occur in ischemic brains. Mouse models of Alzheimer’s disease supported this suggestion that modified tau protein is closely related to amyloid accumulation in Alzheimer’s disease, and that tau protein phosphorylation contributes to amyloid toxicity [8]. In Alzheimer’s disease, the retinoic acid receptor-b succumbs hyperacetylation, resulting in decreased activity of α-secretase and alternative metabolism of the amyloid protein precursor by β- and γ-secretase [93]. In this way, excess amyloid is formed, which then self-aggregates on the surface of neuronal cells, causing their dysfunction and death [93]. Toxic amyloid increases glutamate uptake and inhibits acetylcholine release at synapses, changes in the behavior of these neurotransmitters are a key phenomenon in Alzheimer’s disease and brain ischemia [7,9,16,94,95]. Therefore, much remains to be understood as to what determines the regulation of acetylation, e.g., tau protein and how it contributes to key processes in Alzheimer’s disease and cerebral ischemia. What enzymes are characteristic to acetylate the tau protein, the enzymes that metabolize the amyloid protein precursor and other proteins engaged in the progression of Alzheimer’s disease and cerebral ischemia? What about lysine deacetylases different than sirtuin 1: do they influence the acetylation state in this case of the tau protein participating in neuronal plasticity and survival? Does acetylation of specific lysines, separately or together, affect the phosphorylation status of nearby threonine, serine or tyrosine residues in phosphoproteins participated in learning and memory? Lysine deacetylase activators and inhibitors and acetyltransferase inhibitors should be intensively investigated in preclinical studies for the treatment of Alzheimer’s disease and brain ischemia, diseases for which effective causal treatment is urgently needed. Acetylated tau protein may be a pathophysiological phenomenon that increases the risk of developing Alzheimer’s disease following brain ischemia.

## Figures and Tables

**Figure 1:**
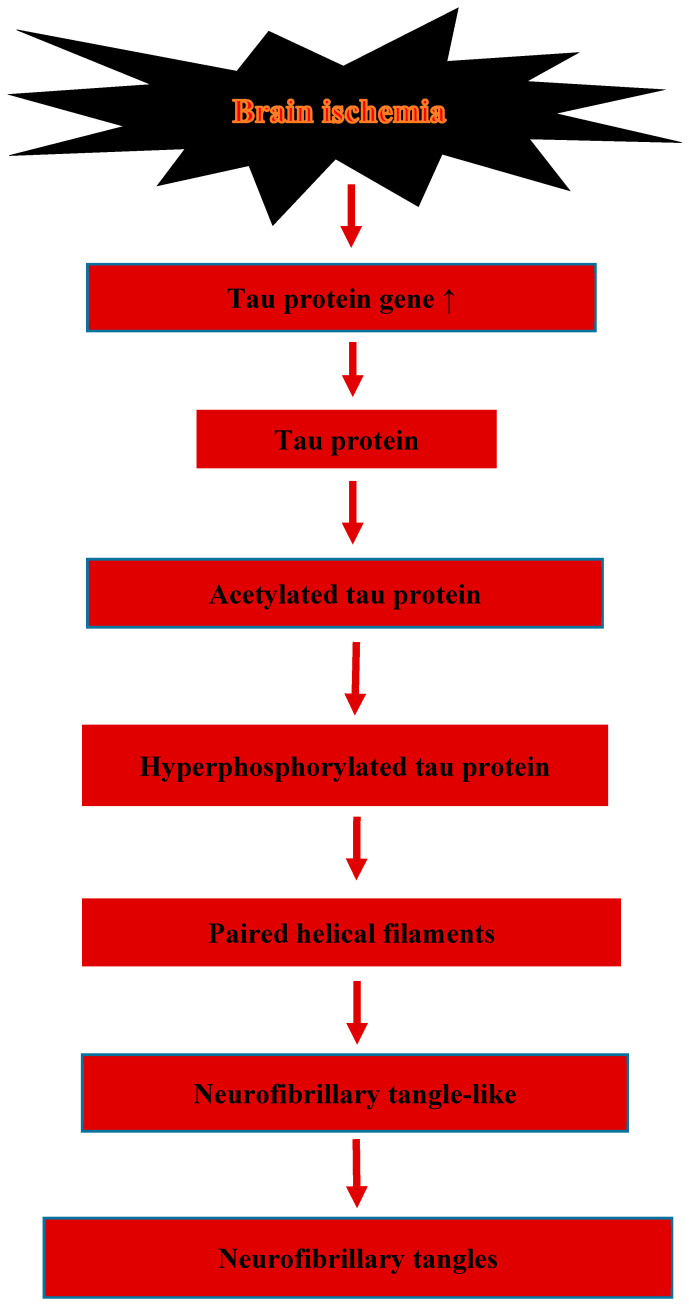
Formation of neurofibrillary tangles after brain ischemia. ↑—increase expression.

**Table 1:** Changes in tau protein gene expression after ischemia.

	Structure	CA 1 Region of Hippocampus	CA 3 Region of Hippocampus
Survival			
2 days		↑↑	↔
7 days		↔	↑
30 days		↔	↑

Expression: ↑↑—increase, ↑—increase, ↔—fluctuation around control value.
